# Coagulation, clipping and closure method: New combined approach to prevent delayed bleeding after gastric endoscopic submucosal dissection

**DOI:** 10.1055/a-2549-9969

**Published:** 2025-04-04

**Authors:** Satoshi Abiko, Haruhiro Inoue, Kei Ushikubo, Kazuki Yamamoto, Yohei Nishikawa, Ippei Tanaka, Naoya Sakamoto

**Affiliations:** 1 378609 Digestive Diseases Center, Showa University Koto Toyosu Hospital, Koto, Japan; 2 Department of Gastroenterology and Hepatology, Hokkaido University Graduate school of Medicine, Hokkaido, Japan

## Introduction

Application of the coagulation, clipping and closure method.Video 1

There are several methods for preventing delayed bleeding (DB) after gastric endoscopic submucosal dissection (ESD), such as the coagulation plus clipping method and the closure method; however, they have not been able to completely prevent DB 1 2 3 . It was hypothesized that DB could be completely prevented through a combination of advanced closure with a coagulation and clipping method. Prevention of DB with this coagulation, clipping and closure (CCC) method is reported here.

**Fig. 1:**
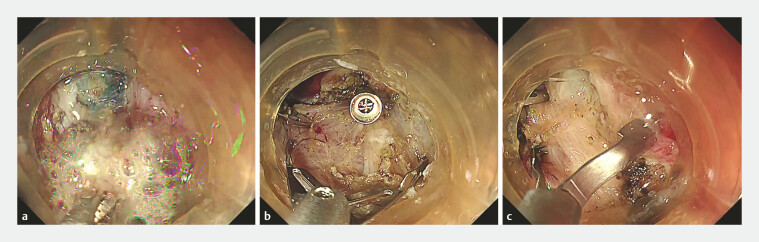
Coagulation, clipping and closure method for preventing delayed bleeding after gastric endoscopic submucosal dissection. **a** First, a coagulation procedure was performed after lesion resection, targeting vessels primarily at the margin of the ulcer base. **b** Next, perforator vessels emerging between the muscle layers and the surrounding muscle layer were clipped using 16-mm or 11-mm reopenable clips (SureClip; Micro-Tech Co. Ltd, Nanjing, China) while sufficient air was suctioned. **c** Complete closure of the residual mucosal defect was achieved using the dead space-eliminating technique with anchor pronged clips (MANTIS clip; Boston Scientific, Marlborough, Massachusetts, United States).

**Fig. 2:**
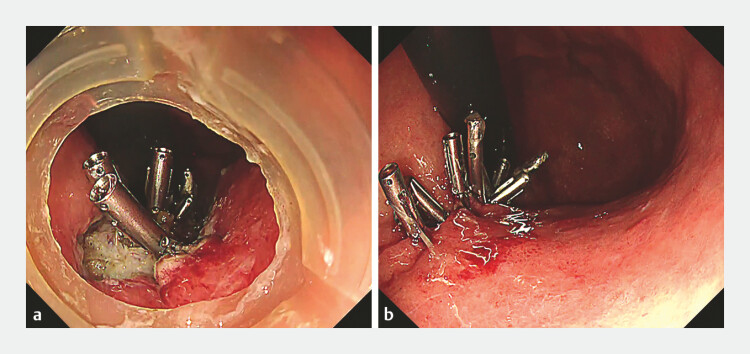
Condition after endoscopic submucosal dissection (ESD). **a** The muscle layer is folded using 16-mm or 11-mm reopenable clips. **b** The ulceration was completely closed using a coagulation, clipping and closure method.

Gastric ESD was performed for an 83-year-old man classified as having a high risk of DB due to oral administration of rivaroxaban and prasugrel. The ulceration followed gastric ESD (30 mm in diameter) in the lesser curvature of the upper body of the stomach. First, a coagulation procedure was performed after lesion resection, targeting vessels primarily at the margin of the ulcer base ( Fig. 1 **a** ). Next, perforator vessels emerging between the muscle layers and the surrounding muscle layer were clipped using 16-mm or 11-mm reopenable clips while sufficient air was suctioned ( Fig. 1 **b** ). As a result, the muscle layer folded. This procedure, inspired by the Origami method 4 , reduces the size of the mucosal defect ( Fig. 2 **a** ). Finally, complete closure of the residual mucosal defect was achieved using the dead space–eliminating technique with anchor pronged clips 5 ( Fig. 1 **c** , Fig. 2 **b** , and Video 1 ).

Bleeding from perforator vessels may occur in areas where wound closure has dehisced; however, it is believed that this type of DB can be prevented by preemptively coagulating and clipping perforator vessels at the base of the ulcer. Preemptively folding the rigid gastric muscular layer, which is prone to dehiscence, using clips may be an effective procedure for preventing subsequent dehiscence.

The CCC method may help reduce risk of DB after gastric ESD; however, further evaluation with a larger number of cases is required to validate its effectiveness.
